# IP-10 dried blood spots assay monitoring treatment efficacy in extrapulmonary tuberculosis in a low-resource setting

**DOI:** 10.1038/s41598-019-40458-0

**Published:** 2019-03-07

**Authors:** Ida Marie Hoel, Melissa Davidsen Jørstad, Msafiri Marijani, Morten Ruhwald, Tehmina Mustafa, Anne Ma Dyrhol-Riise

**Affiliations:** 1 Centre for International Health, Department of Global Public Health and Primary Care, University of Bergen, Bergen, Norway; 2 Department of Clinical Science, University of Bergen, Bergen, Norway; 3 Department of Thoracic Medicine, Haukeland University Hospital, Bergen, Norway; 4 Department of Diagnostic Services, Mnazi Mmoja Hospital, Zanzibar, United Republic of Tanzania; 5 Department of Infectious Disease Immunology, Statens Serum Institut, Copenhagen, Denmark; 6 Department of Infectious Diseases, Oslo University Hospital, Oslo, Norway; 7 Institute of Clinical Medicine, University of Oslo, Oslo, Norway

## Abstract

Treatment efficacy is difficult to evaluate in extrapulmonary tuberculosis (EPTB) patients. Interferon-γ inducible protein (IP-)10 has been suggested as a biomarker for response to treatment. We have investigated if IP-10 from dried plasma spots (DPS) or dried blood spots (DBS) can be used in treatment monitoring of EPTB patients in a low-resource setting of Zanzibar. IP-10 levels in plasma, DPS and DBS samples collected before, during (2 months) and after TB treatment of 36 EPTB patients (6 culture and/or Xpert MTB/RIF positive and 30 clinically diagnosed) and 8 pulmonary tuberculosis (PTB) patients, were quantified by an enzyme-linked immunosorbent assay. There was a high positive correlation between IP-10 measured in plasma and DPS and DBS, respectively. We found a significant decline in IP-10 levels from baseline to end of treatment in plasma, DPS and DBS, both in EPTB and PTB patients. The declines were observed already after 2 months in HIV negative patients. In conclusion, the DPS/DBS IP-10 assay allows for easy and manageable monitoring in low-resource settings and our findings suggest that IP-10 may serve as a biomarker for treatment efficacy in EPTB patients, albeit further studies in cohorts of patients with treatment failure and relapse are needed.

## Introduction

Tuberculosis (TB) is a global health problem with an estimated 10.0 million new cases of active TB and 1.6 million deaths because of TB in 2017^1^. Rapid and robust tests for TB diagnosis and treatments efficacy are needed to achieve global TB control. Although smear microscopy and culture are frequently used to assess treatment responses, the sensitivity of these methods is low in paucibacillary disease^2^, and they also have limited ability to predict treatment outcomes^3–5^. Extrapulmonary TB (EPTB), which accounts for approximately 15–40% of all TB cases^6–9^ with higher numbers in young children and people living with HIV^10–14^, is typically paucibacillary. As smear or culture conversion cannot be used to monitor treatment efficacy in EPTB patients, clinicians must rely on clinical evaluation to detect treatment failure, which is often challenging due to the nonspecific symptoms in EPTB disease. The emergence of multidrug resistant (MDR-)TB^15^ further emphasises the importance of early detection of ineffective treatment or low treatment adherence. Thus, there is a need for new assays to better predict treatment outcomes such as failure, cure and relapse. Biomarkers for early treatment efficacy may also provide surrogate end points in clinical trials for development of more effective and shorter personalized treatment regimens^16^.

Numerous host biomarkers are being investigated as markers for treatment response in TB^17–19^, including the pro-inflammatory chemokine interferon-γ inducible protein (IP-)10, also known as CXCL-10. Several studies show that plasma and serum IP-10 levels decline upon efficient treatment of TB^20–26^. Further, IP-10 is a robust marker expressed at higher levels than many other candidate biomarkers^20,27^, which allows for IP-10 detection using simple test platforms.

Dried plasma spots (DPS) and dried blood spots (DBS) applied on filter paper is a simple and robust method for storage and transportation of blood specimens. Several substances, including IP-10, are stable in DPS and DBS, even when kept at ambient temperatures^27,28^, and IP-10 extracted from DPS and DBS can be quantified using ELISA-based methods^27,29,30^. Moreover, a user-friendly quantitative lateral flow assay for detection of IP-10 from antigen stimulated blood in TB patients has shown promising results^31,32^, indicating that IP-10 based assays, eventually combined with other biomarkers, have the potential to be developed into simple point-of-care (POC) tests.

We have previously shown that IP-10 levels in DPS decline already after two weeks of therapy both in pulmonary TB (PTB) and EPTB^24^. Still, most of the studies on IP-10 as a marker for response to treatment are on PTB and data on EPTB in resource-constrained settings is scarce. The objective of this study was to compare IP-10 obtained from DPS and DBS to IP-10 measured directly in plasma in order to evaluate the performance of the IP-10 DBS assay as a biomarker for response to treatment in EPTB patients in a clinical low-resource setting.

## Results

### Study participants and clinical outcome

**Figure 1:**
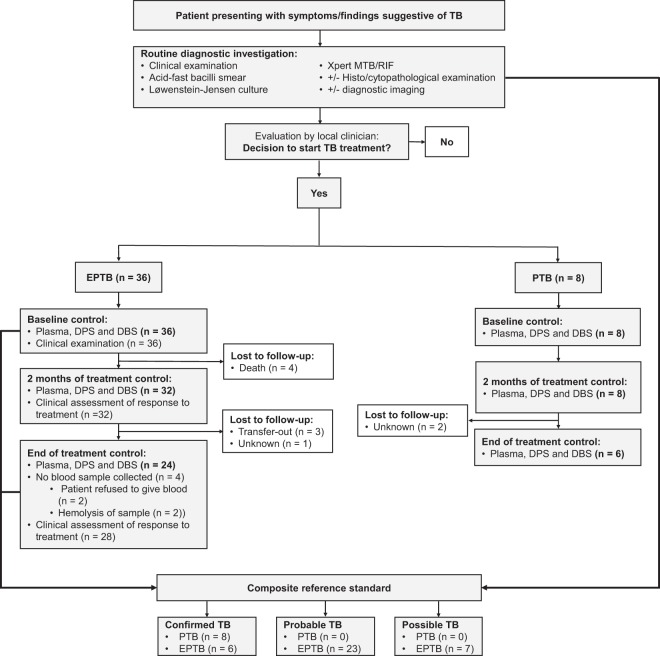
Flow chart of patient inclusion and data collection during the study. Abbreviations: TB, tuberculosis; EPTB, extrapulmonary TB; PTB, pulmonary TB; DPS, dried plasma spot; DBS, dried blood spot.

Eight patients with PTB and 36 patients with EPTB were longitudinally followed during TB treatment (Fig. 1). Clinical characteristics are presented in Table 1. All PTB patients and six EPTB patients had bacteriologically confirmed TB (positive culture and/or Xpert MTB/RIF) and 30 EPTB patients had a clinical TB diagnosis, categorised as a probable TB case (n = 23) and possible TB case (n = 7), based on a composite reference standard^33^. All PTB patients were sputum smear positive at baseline. A drug susceptible *Mycobacterium tuberculosis* strain was detected in 5/10 culture positive samples, whereas drug susceptibility testing was not performed in the remaining five cases. No genotypical rifampicin resistance was detected in the 10 Xpert MTB/RIF positive samples. All PTB patients and 32 EPTB patients received standard TB treatment (isoniazid, rifampicin, pyrazinamide and ethambutol) for 6–10 months, whereas four EPTB patients received modified treatment because of previous PTB or active hepatitis. After 2 months of treatment, 2/8 PTB patients remained sputum smear positive, both of whom demonstrated smear conversion at the follow-up sputum control one month later. A successful treatment outcome was recorded in the National TB program registers for all the PTB patients. All EPTB patients that were evaluated at 2 months showed clinical improvement, except four patients, three of them HIV-infected, who presented with a transient enlargement of lymph nodes, possibly due to paradoxical immune reactions. At the end of treatment, all EPTB patients that completed therapy demonstrated clinical improvement. Blood samples were taken before start of TB treatment, after 2 months and at the end of treatment. Altogether, four patients died, six patients were lost to follow-up and a final blood sample was not obtained from four patients (Fig. 1). Thus, a complete set of samples at three time points were available for six PTB patients and 24 EPTB patients.

**Table 1:** Characteristics of the study participants.

Patient characteristics	EPTB (n = 36)	PTB (n = 8)
Age in years, median (range)	33 (18–67)	34 (20–52)
Female, n (%)	18 (50)	2 (25)
HIV-infected, n (%)	9 (26)^a^	2 (25)
**TB case category, n (%)**		
Confirmed TB	6 (17)	8 (100)
Probable TB	23 (64)	
Possible TB	7 (19)	
**Routine diagnostics (positive/total)**		
Culture (baseline)	3/32	7/7
Xpert (baseline)	5/23	5/5
AFB smear baseline	6/36	8/8
AFB smear 2 months	NA	2/8
AFB smear 5 months	NA	0/7^b^
**Site of infection, n (%)**		
Lymph node	16 (44)	
Pleura	9 (25)	
Abdomen	4 (11)	
Spine	1 (3)	
Lymph node and PTB	2 (6)	
EPTB ≥2 sites/disseminated TB	4 (11)	
**ART status for HIV-infected patients, n (%)**		
ART before start of TB treatment	3 (33)	NA
ART initiated during TB treatment	4 (44)	NA
Patient died before initiating ART	2 (22)	NA

Abbreviations: EPTB, extrapulmonary tuberculosis; PTB, pulmonary tuberculosis; AFB, acid fast bacilli; ART, antiretroviral therapy; NA, not available or performed.

^a^HIV status was unknown for one EPTB patient.

^b^Sputum smear result at 5 months was missing for one PTB patient. This patient was registered with a negative sputum smear at 2 months.

### IP-10 decline corresponds to sputum conversion in pulmonary TB patients

**Figure 2:**
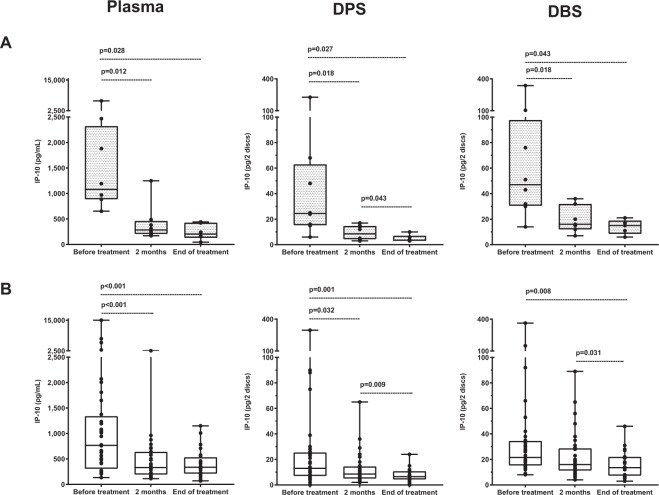
Changes in IP-10 levels in plasma, dried plasma spots (DPS) and dried blood spots (DBS) during tuberculosis (TB) treatment. IP-10 concentrations measured using enzyme-linked immunosorbent assay from (**A**) patients with pulmonary TB before treatment (n = 8), at 2 months (n = 6) and at the end of treatment (n = 6) and from (**B**) patients with extrapulmonary TB before treatment (n = 36), at 2 months of treatment (n = 32) and at completion of 6–10 months of treatment (n = 24). Boxes represent the median and interquartile range, and the whisker show min/max values. Dotted lines indicate statistically significant differences. Statistical significance was evaluated by Wilcoxon Signed Ranks test using a significance level of 0.05.

We first analysed IP-10 levels in the eight patients with confirmed active PTB disease (Fig. 2A). There was a significant decline in IP-10 from baseline to the end of treatment in plasma samples [1081 pg/mL (886–2321) vs. 203 pg/mL (129–427), p = 0.028], DPS samples [25 pg/2 discs (15–63) vs. 4 pg/2 discs (3–7), p = 0.027] and DBS samples [47 pg/2 discs (31–98) vs. 15 pg/2 discs (9–19), p = 0.043]. The decline in IP-10 levels was observed in 8/8 PTB patients already after 2 months of treatment in plasma (p = 0.012), DPS (p = 0.018) and DBS (p = 0.018), and corresponded with sputum conversion for six of the patients.

### Plasma IP-10 decline during TB treatment in extrapulmonary TB

**Figure 3:**
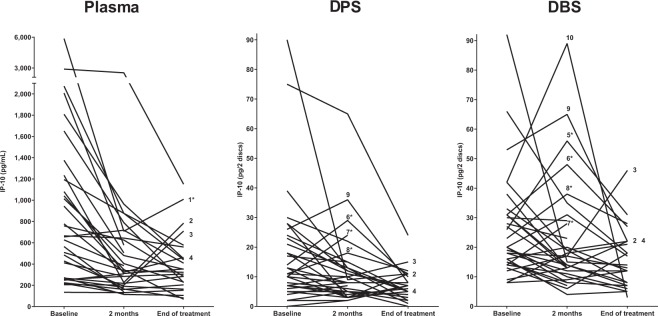
Comparison of IP-10 levels in plasma, dried plasma spots (DPS) and dried blood spots (DBS) for individual extrapulmonary tuberculosis (EPTB) patients during TB treatment. IP-10 levels during 6–10 months of TB treatment in **plasma**, **DPS** and **DBS** in patients with extrapulmonary TB at baseline (n = 32), at 2 months of treatment (n = 32) and at the end of treatment (n = 24). IP-10 levels in baseline samples from the four patients who died shortly after start of TB therapy, are not presented in this figure. ^1^TB pleuritis, ^2^Combined ascites and TB pleuritis, ^3–4^Pregnant during treatment, ^5–7^Persistant or increasing lymph node swelling at 2 months of treatment, ^8–9^Lymph node TB, ^10^Abdominal TB. *HIV-infected patient.

We then analysed the *plasma* levels of IP-10 in the patients with EPTB. There was a significant decline in IP-10 levels from baseline to the end of treatment [660 pg/mL (269–1224) vs. 337 pg/mL (210–535), p < 0.001] (Fig. 2B). As for the PTB patients, the IP-10 decline was observed already after 2 months (p < 0.001). Individual changes in IP-10 levels during TB treatment are presented in Fig. 3. In most EPTB patients, plasma IP-10 levels decreased from baseline to each follow-up time point and corresponded to a good clinical response to treatment. However, markedly increasing plasma IP-10 levels towards the end of treatment were seen in four patients despite good clinical response. Two of these patients were pregnant during treatment (patient 3–4 in Fig. 3), the third patient was HIV-infected (patient 1 in Fig. 3) and the last patient with ascites and pleuritis demonstrated pleural thickening on X-ray at the last visit but was otherwise in general good clinical state (patient 2 in Fig. 3). No intercurrent infections were registered in any of these patients, who all finished treatment successfully. In contrast, only one of four patients that presented with increasing or persistent lymph node swelling at 2 months of therapy showed a corresponding increase in IP-10 levels.

### DBS IP-10 and DPS IP-10 decline during TB treatment in extrapulmonary TB

We found a high positive correlation between IP-10 measured directly in plasma and IP-10 extracted from DPS (r = 0.809, p < 0.001), and DBS (r = 0.767, p < 0.001) in all samples. As in the plasma samples, there was a significant decline in IP-10 levels from baseline to the end of treatment in both DPS [12 pg/2 discs (7–24) vs. 7 pg/2 discs (4–11), p = 0.001] and DBS [20 pg/2 discs (15–31) vs. 14 pg/2 discs (7–22), p = 0.008] (Fig. 2B). However, a significant decrease in IP-10 from baseline to 2 months of treatment was only observed in DPS (p = 0.032) and not in DBS (p = 0.217). In contrast to the plasma samples, a further decline in IP-10 from 2 months to the end of treatment was seen both in DPS (p = 0.009) and DBS (p = 0.031) samples.

Similar patterns of individual changes in IP-10 levels during treatment were observed in the filter paper samples as in the corresponding plasma samples for the majority of patients. However, an apparent IP-10 increase from baseline to 2 months of treatment was seen more often in DPS and DBS filter paper samples compared to plasma samples (Fig. 3). Interestingly, this included three of the four patients who did not demonstrate clinical improvement after 2 months of therapy (patient 5–7 in Fig. 3), as well as two other patients with lymph node TB (patient 8–9 in Fig. 3), one with an intercurrent inflammation, but both with clinical response to TB therapy. Further, a transient and high IP-10 increase in DBS only, was seen in one patient with abdominal TB with good response to treatment (patient 10 in Fig. 3).

### IP-10 levels in various clinical groups of TB patients

In general, there was a tendency to higher IP-10 levels at baseline in PTB patients compared to EPTB patients both in DPS (p = 0.075) and DBS (p = 0.013), but not in plasma (p = 0.114). When EPTB patients with concurrent PTB infection (n = 2) or disseminated TB (n = 4) were excluded from the analysis, the difference in IP-10 levels between the PTB and EPTB patients became even more apparent both for DPS (p = 0.045) and DBS (p = 0.01).

**Figure 4:**
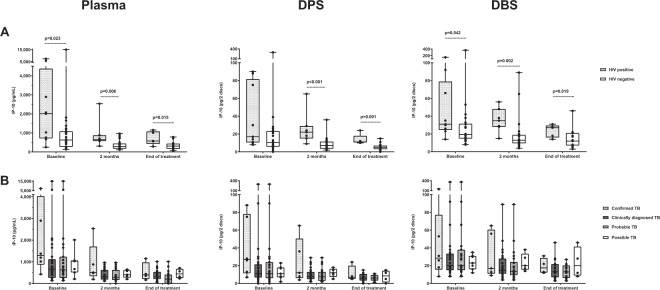
IP-10 levels in different clinical groups of extrapulmonary tuberculosis (EPTB) patients during treatment in plasma, dried plasma spots (DPS) and dried blood spots (DBS). IP-10 concentrations were measured using enzyme-linked immunosorbent assay. EPTB sub-grouped according to (**A**) HIV status; before treatment (n = 35), at 2 months of treatment (n = 32) and at the end of treatment (n = 24), and according to (**B**) TB category; before treatment (n = 36), at 2 months (n = 32) and at the end of treatment (n = 24). HIV status was unknown for 1/36 EPTB patients. Boxes represent the median and interquartile range, and the whisker show min/max values. Dotted lines indicate statistically significant differences. Statistical significance was evaluated by Mann-Whitney U test, using a significance level of 0.05.

Since the vast majority of EPTB patients were clinically diagnosed (83%), we also compared IP-10 levels between patient groups according to TB diagnostic criteria. At baseline, there was a tendency to higher IP-10 levels in plasma (p = 0.071) and DPS samples (p = 0.086) from EPTB patients with confirmed TB diagnosis compared to patients with clinical TB diagnosis (Fig. 4). For those groups large enough to analyse we found no significant differences in baseline IP-10 levels between the various extrapulmonary sites of infection.

### The effect of HIV co-infection on IP-10 levels during TB treatment

In the EPTB group, the IP-10 levels were significantly higher in HIV-infected (n = 9) compared to HIV negative (n = 27) patients at all time points and assessed by all three methods (Fig. 4). In contrast to the joint analyses of HIV-infected and HIV negative EPTB patients, a significant decline in IP-10 at 2 months of treatment was observed not only in plasma (p < 0.001) and DPS (p = 0.011), but also in DBS (p = 0.045) when HIV negative patients were analysed separately. For the HIV-infected EPTB patients, the IP-10 decline at 2 months of treatment was only significant in plasma (p = 0.043), but only few patients were included in these analysis.

## Discussion

In this study, we have evaluated the potential of the DPS/DBS IP-10 assay compared to measuring IP-10 in plasma, for assessing treatment responses in EPTB patients in a clinical setting where diagnostic tools and resources are limited. To our knowledge, this is the first study evaluating IP-10 from DPS/DBS during the first months of TB treatment also in clinically diagnosed EPTB patients. We demonstrate that IP-10 is readily detectable both in unstimulated plasma and DPS/DBS filter paper samples. Further, we show that IP-10 levels decrease upon completion of successful treatment in plasma, DPS as well as DBS, and that the decline is significant already after 2 months of treatment in HIV negative patients.

IP-10 is a pro-inflammatory chemokine expressed by antigen presenting cells, mainly in response to T-cell derived interferon-γ^34^. Increased levels of IP-10 are found in blood, plasma or urine in infections such as HIV^35,36^, hepatitis C^37–40^, bacteremia^41,42^ and TB^43–50^. It has recently been demonstrated that IP-10 secretion during TB disease originates from infected macrophages and multinucleated giant cells located in the granulomas, and early inactivation of the secreted chemokine by antagonist form of IP-10 which binds to CXCR3 but does not induce signalling, may also play a role in the pathogenesis of TB^51,52^. Several studies show that IP-10 declines during efficient treatment of many diseases including TB^25,38,42,53,54^. Limited data also indicates that IP-10 increases in non-cured TB patients from baseline to 2 months of treatment, and in TB patients who relapse or develop active TB disease^25,55^. This makes IP-10 a strong candidate as a biomarker for treatment response although specificity is an issue. In our study, we found a significant decline in plasma IP-10 after 2 months and upon completion of successful treatment, which is consistent with findings in several other investigations^20,22–26^. However, as all of the TB patients in our cohort responded to treatment, we cannot fully evaluate the potential of IP-10 to detect treatment failure in the present study. Further, a significant decline in plasma IP-10 levels has been reported to occur as early as 1–2 weeks after initiation of TB therapy^24,26,54^. We did not include earlier time points for evaluation due to the low-resource setting limiting closer follow-ups, but the DPS/DBS assay may also have potential for earlier evaluation of diagnosis and treatment efficacy.

High levels of IP-10 are observed in a variety of inflammatory diseases and conditions^56–58^. We found markedly increasing IP-10 levels towards the end of treatment despite clinical improvement in four patients. Two of the patients were pregnant during therapy, which is known to cause physiological changes in cytokine profiles^59^, and one patient presented with an unspecified inflammatory swelling in a foot. This is in line with our previous observation that intercurrent disease could cause IP-10 production^24^, and emphasises that IP-10 measured in unstimulated blood specimens is not a specific marker for TB disease and must be interpreted within a clinical context. Likewise, a significantly higher level of plasma IP-10 in HIV-infected patients compared to HIV negative TB patients was observed throughout treatment in our study, which may reduce the specificity of IP-10 based tests in HIV-infected patients. However, we and others have shown that IP-10 levels can differentiate patients with latent TB infection from patients with TB disease irrespective of HIV status^23,26^. With regard to the impact of HIV infection on IP-10 responses during TB treatment, discordant results are reported^20,60^. We observed a decrease in plasma IP-10 during treatment in EPTB patients irrespective of HIV status, which is supported by a recent study demonstrating a decline in serum IP-10 levels during the early phases of treatment in HIV-infected patients^54^. For the DPS and DBS samples in our study, the IP-10 decline was only significant in HIV negative patients, but the HIV patient group is too small to conclude.

Evaluation of treatment efficacy in EPTB patients is important for timely and proper management, as many are started empirically on treatment due to low sensitivity of routine confirmatory tests. Further, smear and culture conversion cannot be used to assess response, underlining that EPTB patients would greatly benefit from new methods for treatment monitoring. We found a tendency to higher levels of pre-treatment plasma IP-10 in bacteriologically confirmed EPTB patients compared to clinically diagnosed patients, which supports the assumption that clinically diagnosed EPTB patients in general have a lower bacterial burden associated with less advanced disease. Still, there was a significant decline in IP-10 both after 2 months and at the end of treatment also for patients defined as *probable TB* cases.

DBS provides a minimally invasive method for blood collection using finger prick blood, and requires no centrifugation for sample processing, making DBS particularly user-friendly in low-resource settings. Our data indicate that the DBS IP-10 method can be used in settings where storage and transport of plasma is not feasible. However, discordant results between the three methods were observed for some patients, mostly HIV-infected. We have no clear explanation to this, but due to a limited number of DPS/DBS per patient, all filter paper samples were assayed as singlets in the ELISA. The use of triplicates in further studies would provide important information about whether variance in the measurements is causing discordant results between methods. Further, apparent stable or weakly fluctuating low levels of plasma IP-10 (<300 pg/mL) throughout treatment were observed in a few EPTB patients despite good clinical response. TB patients represent a heterogenous group of patients, both in terms of infection sites and variation in systemic inflammation. Thus, low systemic plasma IP-10 levels in some patients could reflect low disease activity already at baseline, or compartmentalized production and degradation of cytokines at the site of infection, both limiting the use of the assay in this group of TB patients.

There are some limitations to our study. The small sample size gives reduced power to our results. As we did not have any TB patients with unsuccessful treatment and only followed patients until completion of treatment, the association between IP-10 and treatment failure or relapse cannot be assessed. Further, several factors may influence IP-10 levels and lead to variation; firstly, the EPTB group was heterogenous with various TB localisations, secondly, the number of HIV-infected patients was low, CD4 cell counts were not performed and the anti-retroviral therapy coverage during TB treatment varied according to decisions made by local clinicians. Thirdly, a clinical TB diagnosis is uncertain and elevated IP-10 levels could reflect other diagnoses than TB, although we believe that the use of a composite reference standard for TB diagnosis that includes clinical treatment response makes this less likely. Finally, the time points for sample collection during therapy varied somewhat compared to the original study protocol, as many follow-up controls had to be postponed to make it possible for the patients to participate in the study. For a couple of patients, the baseline sample was collected a few days after start of TB treatment and may not represent the true pre-treatment level of IP-10, as changes in immune responses have been reported to occur as early as one week after start of treatment^26,54^. Accordingly, to measure IP-10 levels few weeks after initiation of therapy could also have provided data on the kinetics of IP-10 in a clinical setting.

To conclude, the present study shows that IP-10 declines during successful treatment of a clinically heterogeneous EPTB population in a TB endemic area. The simple and robust DPS/DBS IP-10 method allows for easy and manageable monitoring in low-resource settings. Our data suggest that IP-10 may serve as a biomarker for treatment responses, albeit further studies including larger cohorts of patients with treatment failures and relapses are needed to confirm this.

## Methods

### Study participants

The study was part of a larger study that was conducted at the tertiary care hospital Mnazi Mmoja Hospital at Zanzibar, Tanzania, from August 2014 to September 2015^33^. Thirty-six patients ≥18 years of age with either clinically diagnosed or bacteriologically confirmed EPTB and 8 smear positive and bacteriologically confirmed PTB patients, were included in the study and longitudinally followed during TB treatment (Table 1). The TB diagnosis and decision to start TB treatment was made by local clinical/medical officers. Exclusion criteria were ongoing or earlier treatment for TB during the last 12 months, or refusal to participate in the study. Clinical assessment and collection of blood specimens for the EPTB group were performed before treatment [median day 0 (5–95 percentile range −7 −+ 8 days)], after the intensive phase of treatment at 2 months [median day 71 (5–95 percentile range 54–120)] and at the end of treatment [median day 175 (5–95 percentile range 149–342)]. Treatment outcome and sputum smear results at baseline, 2 months and 5 months of treatment for the PTB patients were collected from the National TB Program registers.

### Diagnostic TB groups

Based on a composite reference standard^33^, the patients were categorized as confirmed, probable or possible TB cases. Briefly, a confirmed TB case was defined as a culture and/or Xpert positive patient. A culture and Xpert negative patient presenting with one of the following; a positive Ziehl-Neelsen (ZN) smear or radiological findings suggestive of TB or histology/cytology consistent with TB or lymphocytosis on fluid cytology, was defined as a probable TB case if the patient also demonstrated clinical response to treatment or had confirmed concomitant PTB. If clinical response to treatment was unknown (lost to follow-up) and there was no concomitant PTB, the same patient was defined as a possible TB case. A patient presenting with symptoms suggestive of TB, but for whom all tests (culture, Xpert, ZN smear, histo/cytopathology and diagnostic imaging) were negative, was defined as a possible TB case if the patient demonstrated good clinical response to treatment.

### Filter paper sample preparation

Whole blood was drawn into EDTA or CPT tubes (Vacutainer, BD). 2 × 25 μL whole blood was spotted directly onto Whatman 903 filter paper (GE Healthcare) before the remaining whole blood was centrifuged within 30–40 minutes after collection to obtain plasma (EDTA tubes at 3000 × *g* for 10 min at room temperature (RT), and CPT tubes at 1800 × *g* for 20 min at RT). Afterwards, 2 × 25 µL plasma was spotted onto another Whatman 903 filter paper. The remaining plasma was stored at −80 °C. The filter papers were left to dry for 3–4 hours at RT before placed in zip-locked plastic bags with desiccants, and stored at −20 °C. The filter paper cards were transported at room temperature and the plasma samples on dry ice to Bergen, Norway, for analysis.

### Protocol for IP-10 determination in plasma, DPS and DBS samples

Quantification of IP-10 in plasma and filter paper samples was performed in triplicates and singlets respectively using ELISA-based assays as described elsewhere by Aabye *et al*. and Drabe *et al*. respectively^27,30^. For the DPS and DBS samples, 2 filter paper discs of 5.5 mm were punched from the dried plasma or blood spots (1 disc per spot) and stacked horizontally in a filter microtiter plate (Luminex, Merck Millipore) and 80 μL dilution buffer was added per well before the plate was incubated at RT for 1 hour to allow for diffusion-based extraction of the DPS/DBS sample. After incubation, the filter microtiter plate was stacked on top of a capture mAb coated ELISA plate containing 20 μL dilution buffer with detection mAb per well. The stacked plates were centrifuged at 700 × *g* for 10 minutes, to filter the extracted sample into the ELISA plate, and incubated at RT for 1 hour. After incubation, plasma, DPS and DBS ELISA plates were washed and revealed for 30 min at RT, before stopping the reaction and reading at 450 nm with 630 nm reference. Plasma levels were corrected for dilutions, DPS and DBS measurements are presented as pg/2 discs.

### Statistical analysis

All values are presented as median and interquartile range (IQR), unless otherwise stated. Statistical analyses were performed using SPSS Statistics V25 (IBM). Non-parametric statistical methods were applied. Wilcoxon Signed Ranks test was used to evaluate change in IP-10 levels between different time points during treatment in individual patients. For comparison of difference in IP-10 levels in independent groups at baseline, Mann Whitney U test was applied when the groups compared consisted of ≥5 individuals per group. Correlation analyses were performed using Spearman’s Rank Correlation Coefficient. A significance level of 0.05 was used. Graphical presentations were made using Prism V7.03. (Graphpad).

### Ethical considerations

Ethical clearance was obtained from the Regional Committee for Medical and Health Research Ethics, Western-Norway (REK Vest) and the Zanzibar Medical Research and Ethics Committee (ZAMREC) before conducting the study. All methods were carried out in accordance with the relevant guidelines and regulations. Permission to export plasma, DPS and DBS samples out of Tanzania was approved by ZAMREC (Ministry of Health, Zanzibar), Mnazi Mmoja Hospital and the study participants. All participants provided informed written consent.

## Data Availability

The datasets generated during and/or analysed during the current study are available from the corresponding author on reasonable request.
